# Determination of gibberellic acid and abscisic acid in (*Zea mays* L.) (ICA-V305) seeds germinated using dynamic sonication assisted solvent extraction and maceration

**DOI:** 10.1016/j.mex.2022.101821

**Published:** 2022-08-13

**Authors:** Juan David Rivera, Diana Marcela Ocampo-Serna, Raúl Andrés Martínez-Rubio, Yaned Milena Correa-Navarro

**Affiliations:** Departamento de Química, Grupo de Estudios Ambientales en Agua y Suelo, Universidad de Caldas, Calle 65 No. 26-10, Manizales, Colombia

**Keywords:** Phytohormones, Dynamic solvent extraction assisted by sonication (DSASE), High performance liquid chromatographic (HPLC), Green chemistry

## Abstract

Phytohormones are molecules responsible for growth, development, and metabolism regulation in plants. Gibberellic acid (GA_3_) and abscisic acid (ABA) are the main phytohormones involved in seed germination. Notably, it should be a highlight that GA_3_ induces germination, whereas ABA inhibits it. For this reason, it is important to calculate the concentration of these two phytohormones during seeds germination.

Firstly, the maize seeds (MS) were germinated and samples of these were taken at different imbibition times, after that, methanol extracts were obtained using two methods of dynamic solvent extraction assisted by sonication (DSASE) and a traditional extraction method (maceration); finally, to estimate the concentration of GA_3_ and ABA a high performance liquid chromatographic method was used.

The results of this study showed that the three extraction methods used, allowed quantifying GA_3_ and ABA during the maize germination time studied. However, of the two extraction methods employed, DSASE was the best technique because higher concentrations of GA_3_ and ABA were found. Therefore, it is important to continue using this green chemistry methodology for these and other analyses.

• The extraction protocol developed was based on dynamic sonication-assisted solvent extraction.
• The chromatographic method used allowed the simultaneous determination of two phytohormones with different physicochemical properties in maize seeds.
• This methodology offers good sensitivity, linearity, precision, reproducibility and suitable detection and quantification limits.

## Graphical abstract

**Figure:**
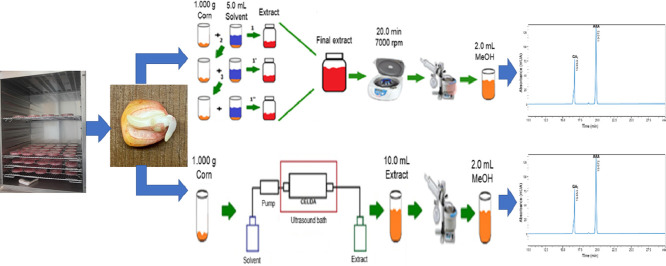

Specifications table

**Table:** 

Subject Area:	Chemistry
More specific subject area:	Analytical chemistry, agrochemical
Method name:	Dynamic sonication-assisted solvent extraction - dynamic (DSASE-D) Dynamic sonication-assisted solvent extraction - static (DSASE-S)
Name and reference of original method:	Dynamic sonication-assisted solvent extraction. *Sanchez C., Ericsson M., Carlsson H., Colmsjö A. and Dyremark E. (2002). Dynamic sonication-assisted solvent extraction of organophosphate esters in air samples. Journal of Chromatography A. 957 (2), 227–234.*
Resource availability:	NA

**Method details**

## Chemicals and reagents

99.0% GA_3_ and 98.5% ABA (Sigma Aldrich, USA), HPLC grade acetonitrile (Fisher Scientific, USA) and analytical grade formic acid (Scharlau, Spain) were used. Water was purified in a Direct-Q system (Millipore, USA).

## Seeds of study

**Fig. 1:**
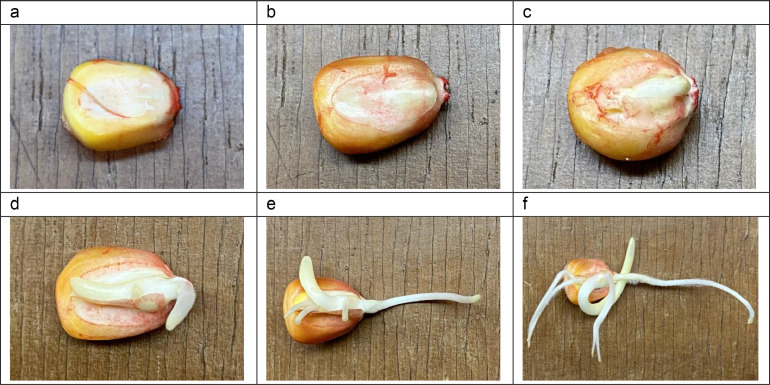
Photographs of maize seeds germinated at different times: (a) 0, (b) 6, (c) 12, (d) 24, (e) 36 and (f) 48 h.

Commercial seeds of maize (*Zea mays* L.) variety ICA-V305 produced by Semillas del Pacífico, were used. The seeds were sieved with a mesh of (6.0 × 6.0) mm, obtaining a volume average of 0.356 ± 0.008 cm^3^ and a mass average per seed of 0.3878 ± 0.0002 g [1]. Maize seeds selected were put in Petri dishes of (100 × 15) mm, into which adsorbent paper moistened with 12.0 ± 0.1 mL of distilled water had been previously placed. After that, the Petri dishes were placed into an incubator at a temperature of 30.1 ± 0.1 °C. Next, after each period of imbibition: 0, 6, 12, 24, 36 and 48 h, maize seeds were removed from the incubator (Fig. 1). Subsequently, maize seeds were crushed in an electric blade mill until a fine powder was obtained, and finally, the powder was stored in a Thermo Scientific freezer at −20 °C to its final use.

## Extraction method

Technique used for phytohormones extraction was as reported by Sanchez et al. [2]. In fact, all samples of powder maize seeds were extracted with methanol-formic acid 5.0% (80:20) as solvent, in addition dynamic sonication-assisted solvent extraction (DSASE) and maceration extraction were used. In particular, DSASE extraction method represents an innovative alternative to maceration because sonication uses acoustic vibrations with frequencies above 20 kHz, as a result of these vibrations, cavitation is produced. For instance, chemical compounds could be mechanically removed from the solid surface and adsorption sites of the sample, because of the collision of the waves produced when cavitation bubbles collapse. In addition, an implosion of cavities causes microenvironments of high temperature and pressure that improve DSASE extraction [3].

For the first procedure 1.0000 ± 0.0005 g of each sample was put in a stainless steel extraction cell, next, the cell was placed inside a Branson 5210 ultrasound bath (Hampton, USA), with an output power of 130 W and a frequency of 40 kHz, in which the temperature was kept at 20–35.0 °C. Finally, the cell was connected to a peristaltic pump to allow continuous solvent flow at 0.5 mL min^−1^. This method was named DSASE-D. The second procedure employed the same quantity of sample and extraction solvent, but after 10.0 min of continuous solvent, flow was stopped for 5.0 min, after that, the extraction was continued for another 10.0 min. This method was named DSASE-S. Each extraction had a duration of 20.0 min and 25 min for the DSASE-D and the DSASE-S, respectively. Finally, extracts obtained were concentrated in a rotary evaporator at 35.0 °C and stored at 4.0 °C until HPLC analysis.

On the other hand, the classical maceration procedure was used, and our experimental set up was based on Zhou et al. [4]. Initially, 1.0000 ± 0.0005 g of sample was placed in a glass vial, next 5.0 mL of methanol-formic acid 5.0% (80:20) was added, after that, the vial was shaken for 1.0 h at room temperature (20.0 °C). Subsequently, solvent was decanted, the supernatant was removed and placed in other vial, while the pellet was extracted two more times with a 5.0 mL of the same extraction solvent. Next, the extracts were combined and centrifuged at 7000 rpm for 20.0 min, Finally, the similar extracts obtained were concentrated in a rotary evaporator at 35.0 °C and stored at 4.0 °C until HPLC analysis.

## Chromatographic method

Phytohormones quantification was carried out employing a high-performance liquid chromatograph Shimadzu, equipped with a degasser (DGU-205), a diode array detector (SPD-M20A), an oven (CTO-10AS VP) and an autosampler (SIL-10AF). In addition, LC-Solution Shimadzu program was used for data analysis (Kyoto Japan).

**Fig. 2:**
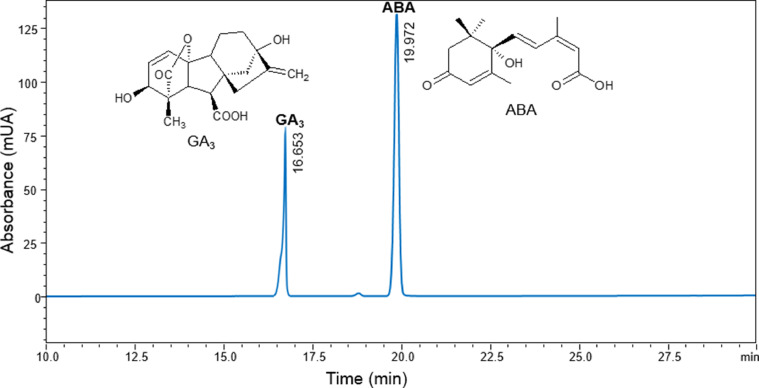
Chromatogram of gibberellic acid and abscisic acid.

For identification and quantification of gibberellic acid and abscisic acid was used a variation of Bhalla et al., procedure [5]*.* In fact, a reversed-phase Supelcosil LC-18-DB column (150 × 4.6 nm, 5.0 µm) was used. Acetonitrile (A) and formic acid 0.01% (B) was employed as mobile phase at a flow rate of 0.5 mL min^−1^, with an 55 min elution program in a linear gradient was set as follows: A:B (10:90%) for 5.0 min, A:B (70:30%) for 40.0 min, A:B (10:90%) for 10.0 min. 10 µL of each sample was injected using an auto-sampling device, moreover, GA_3_ and ABA were detected at a wavelength of 195 nm. In addition, prior to chromatographic analysis, all samples were filtered through 0.2 µm pore PVDF membranes. The experiments were carried out in triplicate and data were expressed as mean ± standard deviation. Fig. 2 shows the chromatogram obtained by high performance liquid chromatography (HPLC) of a standard solution containing 2.0 mg kg^−1^ of GA_3_ and 0.2 mg kg^−1^ of ABA, this displays that the phytohormones were separated efficiently.

**Fig. 3:**
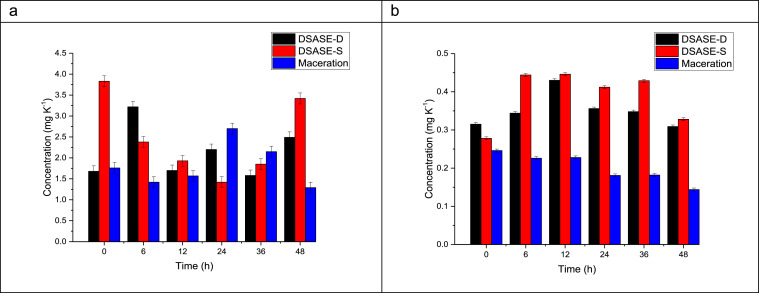
Concentration in maize seeds germinated at different imbibition times using three different extraction methods for (a) gibberellic acid and (b) abscisic acid.

Chromatogram analysis of Fig. 2 allowed us to determine that the chromatographic method used had a selectivity (α) of 1.22, a resolution (R) of 6.57, and a capacity factor (K`) of 10.10 and 12.32 for GA_3_ and ABA, respectively. In addition, after the chromatographic method was achieved, quantification of gibberellic acid and abscisic acid in the methanolic extractions of maize seed was carried out, as a result for that, Fig. 3 shows the concentration of these phytohormones determined in the maize seeds germinated at different imbibition times.

Comparing the extraction methods of maceration, dynamic DSASE, and static DSASE, it was concluded that for ABA the two DSASE methods showed statistically significant differences with respect to the maceration method (p-value 0.0001), while for GA_3_ only the dynamic DSASE method showed differences with respect to the maceration method (p-value 0.0662). Table 1 shows the mean concentrations obtained by the different methods, as well as their standard deviations.

**Table 1:** Statistical comparation of extraction methods used for GA_3_ and ABA quantification employing HPLC.

Method	GA_3_		ABA	
	Concentration (mg kg^−1^)	Uncertainty (mg kg^−1^)	Concentration (mg kg^−1^)	Uncertainty (mg kg^−1^)
Maceration	1.8139	0.5517	0.2467	0.0758
DSASE-D	2.1444	0.5517	0.3517	0.0758
DSASE-S	2.4717	0.5517	0.3906	0.0758

Additionally, Table 1 shows that extraction using the static DSASE technique was the most efficient extraction method employed, yielding a concentration of GA_3_ and ABA of 2.4717 and 0.3906 mg kg^−1^, respectively. The single most marked observation to emerge from the data comparison of the static DSASE extraction was that the concentration of GA_3_ at 12 h, 24 h, and 36 h, did not display statistically significant differences, while at 0 h, 6 h, and 48 h the concentration of GA_3_ showed differences (p-value 0.0053). In addition, the concentration of ABA at 0 h and 48 h displayed statistically significant differences with respect to the other germination times evaluated (*p*-value 0.0708).

Our procedure is a clear improvement of DSASE technique considering that other researchers used constant solvent flow for this method, but in our work a modification was made in particular, the solvent flow was stopped for certain time when pass for sample cell, which results in a mixed methodology: causing a dynamic and static DSASE. Additionally; phytohormones extraction with DSASE is novel, because in the bibliographic review not previous research using this technique for extraction of this metabolites were founded. It is worth noting that DSASE has several advantages when compared to other classical techniques, we can mention the following: low solvent consumption, smaller amount of sample used, reduction of extraction times and slight biomolecules degradation. These and other benefits have led it to become a green chemistry methodology. Finally, the most remarkable result to emerge from the data of this work is that for both hormones analyzed the highest concentration was obtained through the DSASE method [2].

## Validation studies

The chromatographic method was validated in terms of linearity, sensitivity, precision, reproducibility and selectivity [6], the results of these parameters are show in Table 2. To evaluate linearity a calibration curves using seven levels of concentration among 1.00 to 20.00 mg kg^−1^ for gibberellic acid and ten levels of concentration from 0.03 to 2.00 mg kg^−1^ for abscisic acid were obtained. Selectivity was determined through comparison of GA_3_ and ABA retention times (Rt) with samples peaks, moreover, were analyzed absorption spectra of the standards of each phytohormone with absorption spectra of the samples, besides was determined the purity of the phytohormones peaks. Precision was determined as the relative standard deviation ($\%RSD=(s/\bar{x})*100\%$) at three different patron concentrations of GA_3_ (1.0, 10.0, 20.0 mg kg^−1^) and ABA (0.03, 0.50, 2.00 mg kg^−1^). Sensitivity in terms of detection (LOD) and quantification (LOQ) limits was measured. Experimentally LOD was determined by injections of concentrations from 1.0 to 0.2 mg kg^−1^ of GA_3_ and from 0.1 to 0.01 mg kg^−1^ for ABA, these tests revealed the lowest concentration of each phytohormone that showed a detectable chromatographic signal. On the other hand, the limit of quantification (LOQ) was calculated according to the following equation: LOQ = 10/3 LOD [7]. Finally, reproducibility was determined by injecting two concentrations of each phytohormone (4.0 and 16.0 mg kg-1 for GA_3_) and (0.4 and 1.6 mg kg-1 for ABA), at different time intervals for six consecutive days. The average variability using the following equation was calculated.

$$S=\sqrt{\frac{\sum_{i=1}^{6}\left(n_{i}-1\right)S_{i}^{2}}{\sum_{i=1}^{6}\left(n_{i}-1\right)}}$$

**Table 2:** Statistical data of validation.

Validation parameters	Gibberellic acid	Abscisic acid
Linearity (mg kg^−1^)	1.00–20.00	0.03–2.00
Selectivity	No interference was found at the Rt of the phytohormones Absorption spectra of the standard and of the samples were similar Peak purity ˂ 90.00%	
Precision (% RSD)	12.66 (1.0 mg kg^−1^) 2.621 (10.0 mg kg^−1^) 12.39 (20.0 mg kg^−1^)	5.00 (0.03 mg kg^−1^) 1.51 (0.50 mg kg^−1^) 0.48 (2.00 mg kg^−1^)
Limit of detection (LOD) (mg kg^−1^)	0.30	0.01
Limit de quantification (LOQ) (mg kg^−1^)	1.00	0.03
Reproducibility (%)	8.35 (4.0 mg kg^−1^) 8.97 (16.0 mg kg^−1^)	5.79 (0.4 mg kg^−1^) 5.30 (1.6 mg kg^−1^)

## Statistical analysis

The HPLC system has been verified using statistical analysis. Moreover, relationship between the instrumental signal in terms of area and concentration was determined through a simple linear regression employing ordinary least squares method. In addition, the significance (*p* < 0.05) of the data were analyzed through the ANOVA test (analysis of variance) with a confidence interval of 95%, next assumptions of normality; homoscedasticity and independence, of the linear regression were verified. Table 3 shows parameters of the calibration curves with their respective standard deviations, as well as the coefficient of determination of the regression analysis; these data allowed concluding that the regression model for the GA_3_ and ABA quantification was appropriated.

**Table 3:** Parameters of regression analysis for GA_3_ and ABA.

Phytohormone	Intercept $(\beta_{0}$)(mUA*min)	Standard deviation of $\beta_{0}\left(mUA*min\right)$	Slope $\left(\beta_{1}\right)\left(\frac{mUA*min}{mg/kg}\right)$	Standard deviation of $\beta_{1}\left(\frac{mUA*min}{mg/kg}\right)$	Determination coefficient (R^2^)
Gibberellic acid	0.267	2.402	21.607	0.230	99.75
Abscisic acid	0.232	0.381	127.253	0.416	99.97

## Declaration of Competing Interest

The authors declare that they have no known competing financial interests or personal relationships that could have appeared to influence the work reported in this paper.

## Data Availability

Data will be made available on request.
